# Retroperitoneal hemorrhage caused by enoxaparin-induced spontaneous lumbar artery bleeding and treated by transcatheter arterial embolization: a case report

**DOI:** 10.1186/1757-1626-2-9375

**Published:** 2009-12-22

**Authors:** Po-Lin Sun, Yu-Chang Lee, Kuan-Chi Chiu

**Affiliations:** 1 Department of Radiology, E-Da Hospital, I-Shou University, No. 1, Yi-Da Road, Jiau-Shu Tsuen, Yan-Chau Shiang, Kaohsiung County, 824, Taiwan; 2 Department of Radiology, Tainan Municipal Hospital, No.670, Chung-Te Rd. East District, Tainan City, 701, Taiwan

## Abstract

Lumbar artery bleeding with retroperitoneal hematoma is an uncommon life-threatening complication secondary to enoxaparin use. We present a case of 73-year-old Chinese woman with acute retroperitoneal hemorrhage one month following hip surgery, due to enoxaparine. Enoxaparin induced hemorrhage caused by spontaneous rupture of lumbar artery was suspected and treated successfully by transcatheter arterial embolization.

## Introduction

Retroperitoneal hematoma is rare but with increasing incidence due to complications related to interventional procedures. Spontaneous retroperitoneal hematoma is generally seen in patients with anti-coagulation therapy [1]. Retroperitoneal bleeding caused by lumbar artery lesion is rare and mostly related to iatrogenic or trauma [2]. Aneurysm or anticoagulation therapy is the most common causes of non-traumatic bleeding. Only a few cases with enoxaparin-induced spontaneous hemorrhage have been reported in the English literature [3]. Spontaneous retroperitoneal hemorrhage could present as a rare life-threatening emergency with sudden onset of massive bleeding [4].

In this report the patient experienced shock due to a large retroperitoneal hematoma possibly from lesions of lumbar arteries. Emergent CT scan confirmed the diagnosis and immediate angiography and TAE were performed to regain hemodynamic stability.

## Case presentation

In March of 2007, a 73-year-old Chinese woman presented to our ER department due to would discharge and right leg swelling after having received revision of right hip replacement because of recurrent dislocation.

She was admitted to ward under the impression of wound infection and deep vein thrombosis evidenced by serum D-Dimer level of 32.76 mg/L. Her vital signs were stable at the time of admission and the pertinent coagulation data were unremarkable and therefore 30 mg of subcutaneous enoxaparin was given every 12 hours for thromboprophylaxis.

**Figure 1:**
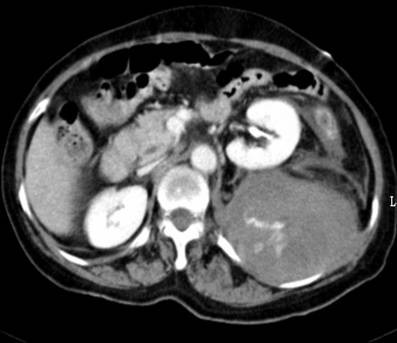
**CT scan demonstrates left retroperitoneal hematoma with contrast medium extravasation**.

**Figure 2:**
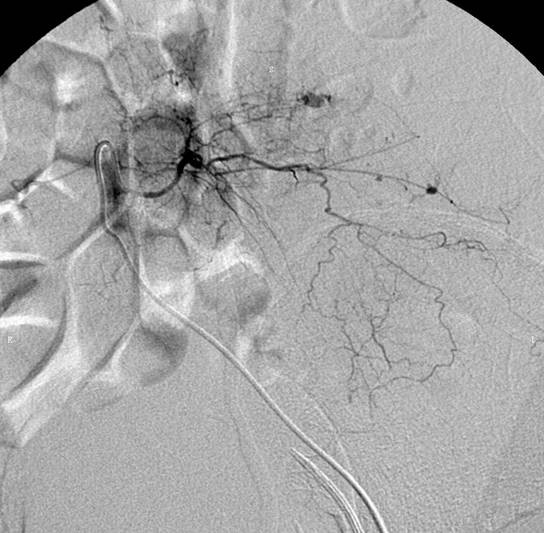
**Selective arteriogram shows contrast medium extravasation from left lumbar artery**.

Sudden onset of left flank pain and cold sweating occurred on the fourth day of admission. Bed-side sonography revealed heterogeneous echogenic fluid collection at the left retroperitoneum. Her blood pressure dropped to 53/33 mmHg with pulse rate of 102 per minute. The administration of enoxaparin was discontinued immediately and the patient was transferred to intensive care unit where she received 4 units of FFP and 12 units and PRBC. After fluid resuscitation, her hemodynamic status remained unstable with pulse rate of 121 per minute and blood pressure 79/56. An emergent CT was performed that showed a large hematoma at the left retroperitoneum (Figure. 1). Emergent angiography was performed which included abdominal aortogram and arteriogram of lumbar arteries at variable levels. The study revealed contrast medium extravasation from left lumbar arteries and branches of left internal iliac artery (Figure. 2). Transcatheter arterial embolization of the lumbar artery and internal iliac artery attempted with deployment of absorbable gelatin sterile sponge. Cessation of bleeding was confirmed on the post embolization angiogram and patient became hemodynamically stable. Two days later, repeat angiography was executed due to unaccountable drop of hemoglobin level and contrast extravasation from a different lumbar artery away from the initial episode was confirmed and embolized again by the same mean. The rest of the hospital course was uneventful and she was discharged three weeks later.

## Discussion

Enoxaparin used in patients with acute coronary syndrome has been increased after several clinical trials [5]. Enoxaparin reportedly has a low incidence of bleeding. Therefore, fatal hemorrhage is actually a rare complication after enoxaparin use. Only two cases of fatal enoxaparin-induced spontaneous retroperitoneal hematoma had been reported in the literature.

Retroperitoneal hematoma can present clinically with groin, flank, abdominal or back pain and it has also been reported as inguinal hernia with appearance of groin swelling [6].

The mainstay treatment of spontaneous retroperitoneal hemorrhage consists of regimen modification or correction of the anticoagulation state, volume resuscitation and transfusion therapy [7]. Amano et al [8] reported that the surgery should be performed immediately in patient with uncontrollable hypotension due to intractable bleeding. But if an initial conservative therapy could control retroperitoneal hemorrhage, a watch-and-wait strategy may be appropriate for the patient. M.A. Tolga et al [9] reported 7 patients with pelvic fracture survived after applying pressure on the bleeding wound to seal off retroperitoneum because retroperitoneum can be treated as one single compartment. Un-clotted wound would lead to continuous bleeding and catastrophic "chimney effect" from the closed retroperitoneal compartment. Hendrickson et al [10] reported that TAE can achieve hemostasis with consolidation of the hematoma, and the subsequent operation could be performed electively.

Endovascular approach is a less invasive treatment of choice that has advantage over surgical intervention which including avoidance of general anesthesia in patients with hemodynamic instability, reduction of blood loss and decrease the risk of releasing the tamponade that may worsen the bleeding.

In our case, unstable hemostasis of the patient and CT scan showed contrast extravasation suggesting active bleeding. We performed TAE to achieve hemostasis, even though rebleeding occurred two days later, a repeat TAE finally secured the bleeding and stabilize the patient successfully.

## Conclusion

Retroperitoneal hematoma caused by enoxaparin should be considered in the differential diagnosis in patients receiving the regimen and experiencing intractable bleeding. Transcatheter arterial embolization appears to be a less invasive and efficacious treatment of choice.

## Abbreviations

TAE: transcatheter arterial embolization.

## Consent

Written informed consent was obtained from the patient for publication of this case report and radiographic images. A copy of the written consent is available for review by the Editor-in-Chief of this journal.

## Competing interests

The authors declare that they have no competing interests.

## Authors' contributions

PS is a major contributor in writing the manuscript; YL is a major contributor in writing the manuscript; KC contributes to the writing and revising of the manuscript. All authors read and approved the final manuscript.
